# The Molecular Determinants of Erythrocyte Removal Impact the Development of Metabolic Dysfunction-Associated Steatohepatitis

**DOI:** 10.2174/0118715303362972241121062515

**Published:** 2024-12-09

**Authors:** Charalampos Papadopoulos

**Affiliations:** 1 Laboratory of Biochemistry, Department of Medicine, Democritus University of Thrace, Alexandroupolis, Greece

**Keywords:** Ferroptosis, erythrophagocytosis, immunometabolism, MASH, erythrocytes, efferocytosis

## Abstract

Metabolic dysfunction-associated steatohepatitis (MASH) is a major cause of a worldwide clinical and financial burden. Despite the tremendous efforts for untangling the molecular mechanisms, there is still a need for defining specific therapeutic targets. In this study, the author will focus on the role of erythrocyte death and hepatic erythrophagocytosis in MASH. Evidence indicates that erythrolysis prior to erythrophagocytosis protects against the development of MASH, while phagocytosis of intact erythrocytes culminates in hepatic inflammation. Furthermore, understanding the balance between erythrolysis and intact erythrocyte engulfment could lead to the development of new strategies for the treatment of MASH.

## 1 INTRODUCTION

Metabolic Dysfunction-associated Steatohepatitis (MASH) is characterized by hepatic lipid accumulation, inflammation, and fibrosis. It affects approximately 4 percent of the human population and is the major cause of liver transplantation. Recently, Rezdiffra was approved for the treatment of MASH, while the main regimen for patients is exercise and diet. The metabolic dysfunction-associated fatty liver disease (MAFLD) leads to a cost of more than 100 billion dollars annually in the U.S. During recent years, this prevalence has increased by more than 50 percent [1-3]. Hence, there is an urgent need to elucidate potential molecular pathomechanisms that are yet unknown and define specific therapeutic targets.

During obesity, the adipose tissue capacity to store fatty acids is surpassed, leading to the formation of bioactive lipids that drive systemic inflammation [4]. Eventually, this inflammatory network impacts the liver, rendering it unable to handle lipid accumulation. Circulating metabolites also impact the hepatic microbiota, which causes epigenetic modifications that drive metabolic regulation [5]. These changes give rise to hepatic lipotoxicity and glucotoxicity, which influence the normal functioning of various cell types, including hepatocytes, macrophages, natural killer cells, T lymphocytes, innate lymphoid cells, neutrophils, eosinophils, platelets, and hepatic stellate cells [6, 7].

Importantly, both lipotoxicity and glucotoxicity, the hallmarks of MASH, also impair erythrocyte function [8, 9]. Erythrocytes are the main iron-containing cells, and during extended erythrocyte damage, the liver undertakes the bulk of erythrophagocytosis and iron storage [10]. The discovery of iron-dependent programmed lytic cell death, ferroptosis, in MASH has sparked increased interest in erythrophagocytosis [11]. In this editorial, the author will try to highlight the pivotal role of erythrocyte death and phagocytosis in the development and treatment of MASH and discuss the implicated molecular mechanisms.

## 2 DISCUSSION

Otogawa *et al.* [12] reported marked erythrocyte damage and hepatic erythrophagocytosis during MASH. In turn, phagocytosis of damaged erythrocytes was followed by upregulated inflammatory markers in the liver. This event was attributed to iron accumulation in macrophages. Interestingly, lipid peroxides, the main marker of ferroptosis, were augmented in both the liver and serum. In addition, increased heme oxygenase-1 levels were reported in hepatic macrophages of MASH animal models. However, since the term ferroptosis had not been coined at the time, the contribution of ferroptosis to hepatic inflammation was not evaluated. Accordingly, Park *et al.* [13] recently reported that augmented erythrophagocytosis in the liver of MASH animals leads to hepatocyte and Kupffer cell ferroptosis, culminating in inflammation. Indeed, similar results have been reported for atherosclerosis [14]. On a molecular level, phagocytosed erythrocytes, after digestion by hepatic macrophages, give rise to an intracellular heme burden. Heme is then metabolized to biliverdin, carbon monoxide, and Fe^2+^, and accumulation of iron then drives ferroptosis and subsequent inflammation.

However, numerous studies have indicated that erythrophagocytosis is anti-inflammatory.  More  specifically,  Pfefferlé *et al.* [15] showed that erythrophagocytosis preceded by erythrolysis in the liver drives the development of a macrophage subset that is anti-inflammatory, exerts continual erythrophagocytosis, and presents a distinct gene expression profile in comparison to the M1 and M2 macrophages. Interestingly, Pfefferlé *et al.* [15] showed that erythrophagocytosis inhibits the development of MASH.

These contradictory results with regard to the role of erythrophagocytosis in MASH initially could trigger confusion. Nevertheless, it seems that the mode of erythrocyte death and phagocytosis determines the role of erythrophagocytosis. In particular, the study of Pfefferlé *et al.* [15] used antibodies against erythrocyte epitopes to induce erythrocyte lysis prior to erythrophagocytosis. This led to the activation of Nrf-2. Mechanistically, heme was incorporated in hepatic macrophages intracellularly by heme-protein complexes and erythrophagocytosis. On the contrary, in MAFLD, where anti-erythrocyte antibodies were not used, erythrophagocytosis was triggered by phosphatidylserine exposure [12] and CD47 loss [16]. Moreover, phosphatidylserine exposure is possibly recognized by various opsonins, such as lactadherin [12], calreticulin, and mannose-binding lectin, but probably not thrombospondin-1 (unpublished data). The generation of the pro-phagocytic signals is mediated by oxidative stress [12], interaction with platelets [13], sphingosine accumulation [16], and hydrolysis of sphingomyelin [17]. Hence, several metabolic steps precede the phosphatidylserine exposure or CD47 loss that drive erythrophagocytosis in MASH. These steps are possibly absent during antibody-mediated erythrophagocytosis.

This data indicate that the mode of death dictates the effect of erythrophagocytosis on inflammation and MASH. However, heme-induced activation of Nrf-2 could also be activated by intact erythrophagocytosis. Importantly, Otogawa *et al.* [12] also reported a mild *ex vivo* lysis of erythrocytes. Thus, the question arises: How, then, erythrophagocytosis in MASH leads to the activation of different intracellular signaling pathways in comparison to antibody-mediated erythrophagocytosis? It is true that the impact of heme and its metabolites on inflammation is largely affected by their levels. More specifically, when there is a mild increase of intracellular heme, its effect is anti-inflammatory, but when the levels rise significantly, there is mainly a pro-inflammatory effect due to over-activation of the intracellular signaling molecules involved, such as Nrf-2 [18].

This leads to the question of how the mode of erythrocyte death affects the levels of heme. Accordingly, Santarino and Vieira [19] showed that the mode of erythrocyte death can largely affect the rate of erythrophagolysosome formation. In particular, when erythrophagocytosis was mediated by erythrocyte ageing and hence programmed cell death, the fusion of erythrophagosome with the lysosomes was delayed by contrast to antibody- or complement–mediated erythrophagocytosis. In this case, it is possible that the increased rate of erythrophagocytosis, along with the delayed erythrophagolysosome formation, could result in inflammation [20]. On the other hand, during antibody-mediated erythrophagocytosis, heme would increase transiently due to a well-preserved balance between erythrophagocytosis and erythrocyte degradation in the lysosomes. Indeed, antibody- mediated erythrophagocytosis has been shown to induce a macrophage metabolic profile characteristic of the M2 (anti-inflammatory) macrophage subset [21]. In addition, a higher hemolysis rate would result in higher heme-protein complexes endocytosed to the macrophages, thus allowing for the phagocytosing cell to adapt to an augmented heme load before erythrophagocytosis.

The next question is what drives the delayed erythrophagosome-lysosome fusion when erythrophagocytosis is preceded by programmed erythrocyte death. It has been shown that ceramide, a lipid molecule implicated in the erythrocyte programmed cell death, drives lysosomal membrane permeabilization, which then leads to slower fusion with the phagosome [22]. On the contrary, when erythrophagocytosis is caused by antibody- or complement-anchoring to erythrocytes, these metabolic alterations on the erythrocyte are not manifested since erythrocyte removal is mediated by antibodies or complement. This mechanism would explain the contradiction between Otogawa *et al.* [12] and Pfefferlé *et al.* [15].

Overall, these studies underscore the dual role of erythrocytes in MASH and add another cell type to the cellular and molecular basis of the disease. Importantly, the connection between erythrocyte metabolism, erythrocyte death, iron handling, and hepatic inflammation sheds light on a unique aspect of immunometabolism.

However, although the results of the research groups mentioned are very interesting, there are some limitations that should be taken into consideration. The study of Otogawa *et al.* [12] used rabbits, whereas Pfefferlé *et al.* [15] used mice. More importantly, Otogawa *et al.* [12] used animals that were fed a high-fat diet for 8 weeks, while Pfefferlé *et al.* [15] used a high-fat diet of 12 days for their animals. Finally, it is not possible to compare the hemolysis index in the *in vivo* environment between the two modes of erythrocyte death.

The therapeutic potential of erythrolysis-accompanied erythrophagocytosis is, however, difficult to evaluate since intravascular hemolysis has been shown to induce hepatic steatosis [23]. This underscores the dual role of heme, which exerts contradictory effects when acting extracellularly or intracellularly. This contradiction is, in part, explained by the extent of hemolysis. When hemolysis is low, then the levels of haptoglobin and hemopexin could be sufficient to deliver intracellular hemoglobin and heme, respectively; however, when hemolysis is excessive, then their levels would not be sufficient to neutralize the extracellular action of heme.

## CONCLUSION

Based on this information, it is possibly postulated that erythrocyte malfunction in MASH leads to hepatic erythrophagocytosis and macrophage ferroptosis. Due to the ferroptosis-induced membrane damage, inflammation would ensue. However, extended erythrocyte removal accompanied by antibody-mediated opsonization would prevent the development of MASH. So far, intracellular heme recognition and the rate of erythrophagolysosome formation have been identified as the main molecular mechanisms governing the effect of erythrophagocytosis on metabolic hepatic inflammation. More studies are needed to elucidate the contribution of erythrophagocytosis in the development and treatment of the disease. The use of erythrolysis as a therapeutic means and the use of erythrocyte-programmed death as a therapeutic target merit further investigation. In particular, analyzing the particular molecular characteristics of the erythrocytes of the patients and studies of the effect of patient-derived erythrocytes on macrophages *ex-vivo* could be the first step for better understanding their role in human MASH.

## LIST OF ABBREVIATIONS

CD47: Cluster of Differentiation 47
MAFLD: Metabolic Dysfunction-Associated Fatty Liver Disease
MASH: Metabolic Dysfunction-Associated Steatohepatitis
